# Immunocytochemical Analysis of the Wall Ingrowths and Cell Wall Microdomains in the Digestive Glands of Venus’ Flytrap

**DOI:** 10.3390/ijms27031193

**Published:** 2026-01-24

**Authors:** Bartosz J. Płachno, Małgorzata Kapusta, Marcin Feldo, Piotr Stolarczyk, Piotr Świątek

**Affiliations:** 1 Institute of Botany, Faculty of Biology, Jagiellonian University, 9 Gronostajowa St., 30-387 Kraków, Poland; 2 Bioimaging Laboratory, Faculty of Biology, University of Gdańsk, 59 Wita Stwosza St., 80-308 Gdańsk, Poland; 3 Department of Vascular Surgery and Angiology, Medical University of Lublin, 16 Staszica St., 20-081 Lublin, Poland; 4 Department of Botany, Physiology and Plant Protection, Faculty of Biotechnology and Horticulture, University of Agriculture in Kraków, 29 Listopada 54 Ave., 31-425 Kraków, Poland; 5 Institute of Biology, Biotechnology and Environmental Protection, Faculty of Natural Sciences, University of Silesia in Katowice, 9 Bankowa St., 40-007 Katowice, Poland

**Keywords:** carbotrace, carnivorous plants, cell wall, cell wall microdomains, digestive glands, *Dionaea muscipula*, Droseraceae, hemicelluloses, pectic homogalacturonan, xyloglucan, xylan

## Abstract

The digestive gland of Venus flytrap consists of various types of specialized cells. Secretory cells form two layers: the first is a more external outer layer and the second is an internal layer that is connected to stalk cells. Our goal was to check whether the position/location of cells is essential in terms of cell wall composition (whether cell wall microdomains exist). We also focused on the structure of cell wall ingrowths in secretory cells. To achieve this, the localization of the cell wall components in the cell walls of gland cells was performed using the immunolabeling technique and confocal microscopy. It has been found that cells within the gland head are not equal. Their location determines the composition of their cell walls in terms of the presence of various epitopes. The cell walls of the secretory cells in the outer layer were deficient in epitopes recognized by antibodies, including JIM5 (low methylesterified homogalacturonans), CCRC-M38 (low methylesterified homogalacturonans), LM5 (galactan), and CCRC-M48 (xyloglucan), which contrasted with the cell walls of the cells in the inner layer. In terms of the occurrence of pectic homogalacturonans, cell wall ingrowths constitute cell wall microdomains. The digestive glands of *Dionaea muscipula* exhibit pronounced cell wall microdomain organization, with distinct distributions of pectins, hemicelluloses, and arabinogalactan proteins across different glandular layers. These compositional differences reflect functional specialization in secretion, absorption, and structural support.

## 1. Introduction

### 1.1. Carnivorous Plants

Carnivorous plants are a specialized group of mixotrophic angiosperms that have evolved morphological, physiological, and biochemical adaptations, enabling them to capture and digest animal prey, primarily arthropods, to supplement their nutritional requirements [1,2,3,4,5]. They have a polyphyletic origin and have evolved several times independently in various angiosperm lineages [6]. Adaptations for carnivory are typically favored in sunny, nutrient-poor habitats characterized by acidic, waterlogged soils or oligotrophic waters, where the availability of nitrogen, phosphorus, and other essential minerals is severely limited [7,8,9].

**Figure 1:**
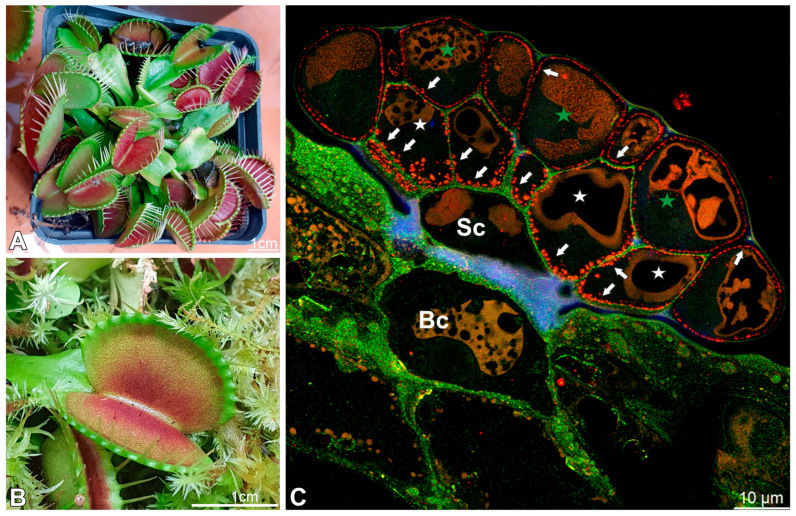
The plant *Dionaea muscipula* and structure of its digestive gland; (**A**,**B**) cultivated *Dionaea muscipula*. (**C**) Cross-section through the gland showing specialized cell types: basal cell (Bc), stalk cell (Sc), secretory cells (white star) of the inner layer, glandular cells of the outer layer (green star); double labeling of cells with CCRC-M38 antibody (a fully de-esterified HGs, green color), and JIM14 antibody (AGPs, the intensive red color), autofluorescence of cutin-impregnated cell walls (blue color), note cell wall ingrowths (arrows).

The hallmark features of carnivorous plants include modified leaves or, less commonly, inflorescence that forms trapping structures, plant enzymatic or symbiont-assisted digestion of prey, and subsequent absorption of released nutrients. Trapping mechanisms are diverse and can be broadly categorized into several functional types [1,10]: active snap traps (*Dionaea muscipula* J. Ellis (Figure 1A,B), *Aldrovanda vesiculosa* L.) [11,12,13], pitfall traps (e.g., *Nepenthes*, *Sarracenia*) [14], adhesive flypaper traps (e.g., *Drosera*, *Pinguicula*, *Byblis*, *Triantha*) [12,15,16,17,18,19], suction traps (*Utricularia*) [12,20,21,22], and eel-trap-like mechanisms (*Genlisea*), e.g., [23,24,25].

The key component of the traps are glands that provide the lytic enzyme cocktail (enzymes such as proteases, phosphatases, chitinases, and others) and absorb nutrients from the digested remains of prey [1,26,27,28,29]. The carnivorous plant glands constitute an elegant, inducible, and tractable in vivo system in which to dissect coupled secretion and absorption dynamics, cell wall remodeling, signaling integration, and transporter deployment, e.g., [30,31,32,33,34,35].

Moreover, the digestive glands of *Dionaea muscipula* J. Ellis (known as Venus flytrap) (Figure 1A,B) provide a paradigmatic system for investigating the rapid induction of secretory and absorptive programs in plant epidermal cells, making them an attractive model for integrative genomic, physiological, structural, and molecular studies [36,37,38]. Mechanical stimulation of trigger hairs, combined with subsequent prey-derived chemical cues, initiates rapid electrical and jasmonate-mediated signaling cascades [33,39], orchestrating a temporally resolved transcriptional reprogramming. Early-response genes encode signaling components and transcription factors, whereas later waves induce hydrolytic enzymes, membrane transporters, and vesicle trafficking machinery [40]. At the cellular level, activated gland cells undergo pronounced ultrastructural remodeling, including expansion of the endoplasmic reticulum and Golgi apparatus, modifications of cell wall porosity, alterations of the vacuolar system, and changes in the occurrence of arabinogalactan proteins (AGPs). These changes reflect a functional switch from a quiescent barrier state to an active secretory (formation of a “green stomach”) and later absorptive phase [35,41,42,43]. Complementary proteomic and metabolomic analyses of digestive fluid and gland tissue have defined the enzymatic cocktail and dynamic small-molecule milieu mediating prey digestion and nutrient release, highlighting key chitinases and proteases [44,45,46,47,48]. Later, nutrients absorbed by the glands [49] are redistributed through the trap and petiole vascular system to support the development of new traps [50].

Recently, Kreuzer et al. [51] showed that prey behavior in the trap activates the endocrine system: mechano- and chemosensors translate the information on the prey’s nature, size, and activity into jasmonate-dependent lytic enzyme secretion. These authors also found that the alterations in *Dionaea*’s metabolism depend on both the type of substance and its amount. Despite enormous progress in understanding the functioning of both the glands and the Venus flytraps themselves, many problems still remain to be solved. Some previously published findings, such as the documentation of “exocytotic vesicle fusion” in the paper by Scherzer et al. [32], may warrant further verification. Hence, there is a need for additional research on organelle dynamics and the processes of exocytosis and endocytosis in Venus flytrap’s digestive glands.

### 1.2. Role of Cell Wall Components in Wall Properties and Transport

The plant cell wall is a dynamic, composite structure that defines cell shape, provides mechanical support, mediates intercellular communication, and regulates transport of solutes and water. The interplay of its major components determines its functional properties: cellulose microfibrils, hemicelluloses, pectins, but also the structural proteins (including extensins, glycine-rich proteins, proline-rich proteins, solanaceous lectins, and arabinogalactan proteins) and, in some tissues, lignin and structural phenolics [52,53,54]. Cellulose microfibrils provide tensile strength and rigidity, forming a scaffold that constrains cell expansion. In contrast, hemicelluloses crosslink microfibrils, modulating wall flexibility and porosity [55,56]. Pectins, rich in galacturonic acid residues, control wall porosity, hydration, and ion-binding capacity, thereby influencing apoplastic transport and diffusion of small molecules [57]. The degree of pectin methyl-esterification and calcium crosslinking modulates wall stiffness and pore size, directly affecting the movement of solutes, including nutrients and signaling molecules, through the apoplast [58]. Structural proteins, including extensins and arabinogalactan proteins (AGPs), contribute to wall architecture and can serve as binding sites for ions or polysaccharides, indirectly regulating transport and cell–cell communication [59].

Cell wall properties also determine the kinetics and specificity of facilitated and passive transport across the apoplast. In tissues with transfer cells, for example, wall ingrowths increase the plasma membrane surface area while maintaining selective apoplastic barriers, thereby enhancing nutrient uptake efficiency [60,61,62]. Offler and Patrick [63] demonstrated that transfer cells are strategically located in regions of high nutrient flux, such as the phloem and developing seeds, highlighting their central role in nutrient allocation. Similarly, in xylem and phloem tissues, cell wall composition influences hydraulic conductivity and solute loading/unloading dynamics [64]. Modulation of wall components during development, stress, or pathogen attack allows plants to fine-tune both mechanical properties and transport processes in response to environmental cues [65,66]. Understanding the molecular contributions of wall polymers and their interactions is essential for elucidating how cell walls coordinate structural integrity with selective permeability and signaling, providing a foundation for engineering crops with improved nutrient transport and stress resilience.

### 1.3. The Aim

While we have analyzed arabinogalactan proteins (AGPs) in the glandular cells of *Dionaea muscipula* [67], there has been no analysis of other components of the cell wall of these digestive glands to date. Our goal was to fill this gap and analyze the presence of cell wall components such as hemicelluloses and pectic homogalacturonans, with particular attention paid to cell wall ingrowths. In particular, the digestive glands have been studied in detail in *Aldrovanda vesiculosa* [67], a sister species to *Dionaea muscipula* [68,69]. Another important aim was to determine whether cell wall microdomains are present in the digestive glands of *Dionaea muscipula*, to check whether the location of glandular cells affects the composition of their cell walls.

## 2. Results

### 2.1. Gland Structure

Each gland consists of basal cells, two stalk (endodermoid) cells, and numerous secretory cells, which form two distinct layers: an outer layer and an inner layer. The cells from the outer layer have contact with the trap environment and cells from the internal layer. Cells from the internal layer mediate between cells from the first layer and stalk cells (Figure 1C). This distinction between layers is essential because, as we show below, there are differences between these cells.

### 2.2. Pectic Homogalacturonan and Other Pectic Polysaccharide Distribution

**Figure 2:**
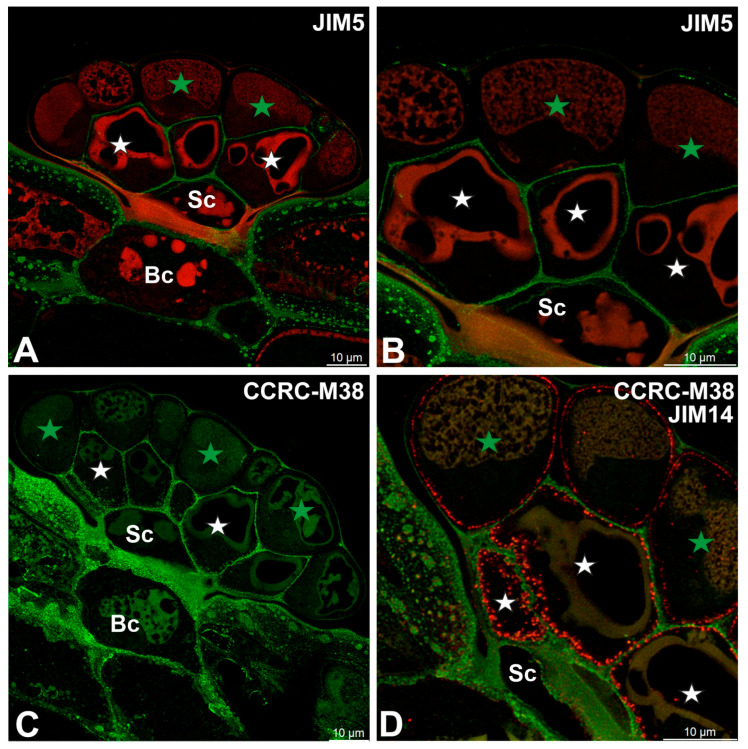
Pectic homogalacturonan (low methylesterified and fully de-esterified HGs) detected in the digestive gland of the *Dionaea muscipula*, (intense green color—signal of antibody, red-brown color—autofluorescence) basal cell (Bc), stalk cell (Sc), secretory cells (white star) of the inner layer, glandular cells of the outer layer (green star). (**A**,**B**) Labeling of cells with JIM5 (low methylesterified HG). (**B**) is an enlarged part of (**A**). (**C**) Labeling of cells with CCRC-M38 antibody (a fully de-esterified HGs). (**D**) Double labeling of cells with CCRC-M38 antibody (a fully de-esterified HGS) and JIM14 antibody (AGPs, shown in intensive red color).

Low-methylesterified homogalacturonans (HGs), detected with the JIM5 antibody, were present in the cell walls of basal cells (Figure 2A,B). Within the glandular head, a pronounced spatial differentiation was evident: cell walls of glandular cells in the inner layer showed clear JIM5 labeling, whereas outer periclinal and anticlinal walls of cells from the outer layer either lacked these epitopes or exhibited only weak signals (Figure 2B). JIM5 labeling was largely absent from cell walls modified by cutinization. A broadly similar distribution pattern was observed for fully de-esterified HGs detected with the CCRC-M38 antibody (Figure 2C,D). In contrast to JIM5, however, CCRC-M38 labeling additionally revealed the presence of fully de-esterified HGs within cell wall ingrowths of glandular cells from the inner layer. Comparison with AGP labeling using the JIM14 antibody showed that cell wall ingrowths were more abundant than indicated by CCRC-M38 labeling alone, suggesting a broader association of arabinogalactan proteins with these wall specializations (Figure 2D).

**Figure 3:**
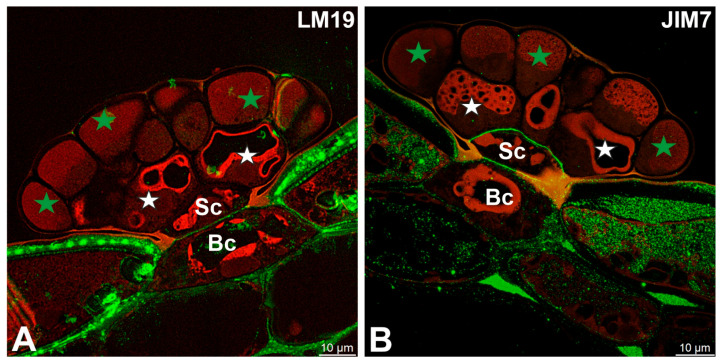
Pectic homogalacturonan (low methylesterified and high methylesterified HGs) detected in the digestive gland of the *Dionaea muscipula*, (intense green color—signal of antibody, red-brown color—autofluorescence) basal cell (Bc), stalk cell (Sc), secretory cells (white star) of the inner layer, glandular cells of the outer layer (green star). (**A**) Labeling with LM19 antibody (low methylesterified HG). (**B**) Labeling with JIM7 antibody (high methylesterified HG).

LM19 (low methylesterified HG) was observed in the cell walls of basal cells (Figure 3A). A fluorescence signal from highly esterified HGs (detected by JIM7) was observed in cell walls of basal cells and stalk cells (Figure 3B).

**Figure 4:**
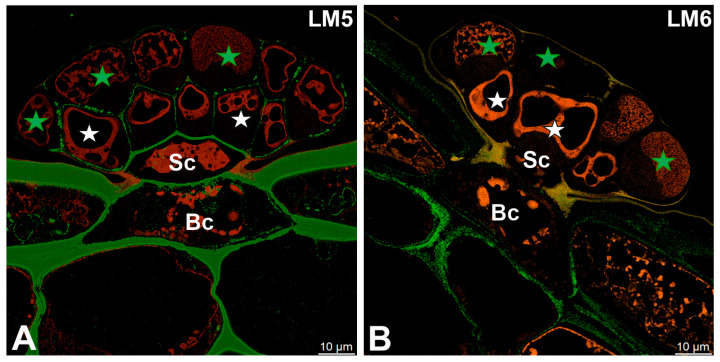
Pectic polysaccharide detected in the digestive gland of the *Dionaea muscipula*, (intense green color—signal of antibody, red-brown color—autofluorescence) basal cell (Bc), stalk cell (Sc), secretory cells (white star) of the inner layer, glandular cells of the outer layer (green star). (**A**) Labeling with LM5 antibody (pectic polysaccharide beta-1,4-galactan). (**B**) Labeling with LM6 antibody (pectic polysaccharide alpha-1,5-arabinan).

The pectic polysaccharide β-1,4-galactan, detected with the LM5 antibody, was predominantly localized to the cell walls of basal and stalk cells (Figure 4A). Within the glandular head, a distinct spatial differentiation was observed: cell walls of glandular cells in the inner layer exhibited clear LM5 labeling, whereas those of the outer layer showed markedly weaker signals (Figure 4A). In contrast, the pectic polysaccharide α-1,5-arabinan, recognized by the LM6 antibody, was confined to the cell walls of basal cells and was not detected in secretory cells (Figure 4B).

### 2.3. Hemicellulose Distribution

**Figure 5:**
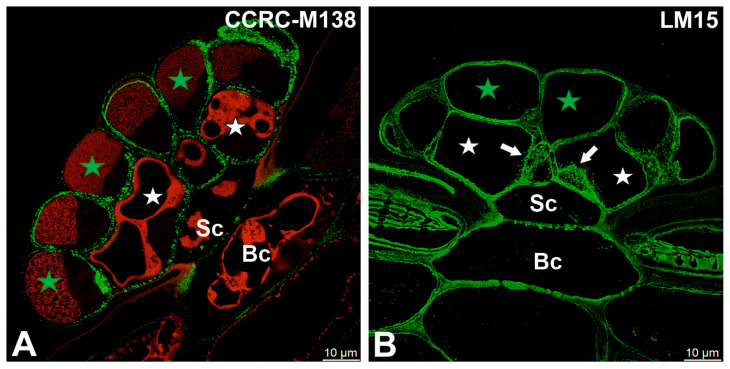
Hemicelluloses detected in the digestive gland of the *Dionaea muscipula*, (intense green color—signal of antibody, red-brown color—autofluorescence) basal cell (Bc), stalk cell (Sc), secretory cells (white star) of the inner layer, glandular cells of the outer layer (green star), cell wall ingrowths (white arrows). (**A**) Labeling with CCRC-M138 (which recognizes the glycan group of Xylan-6). (**B**) Labeling with LM15 (xyloglucan).

**Figure 6:**
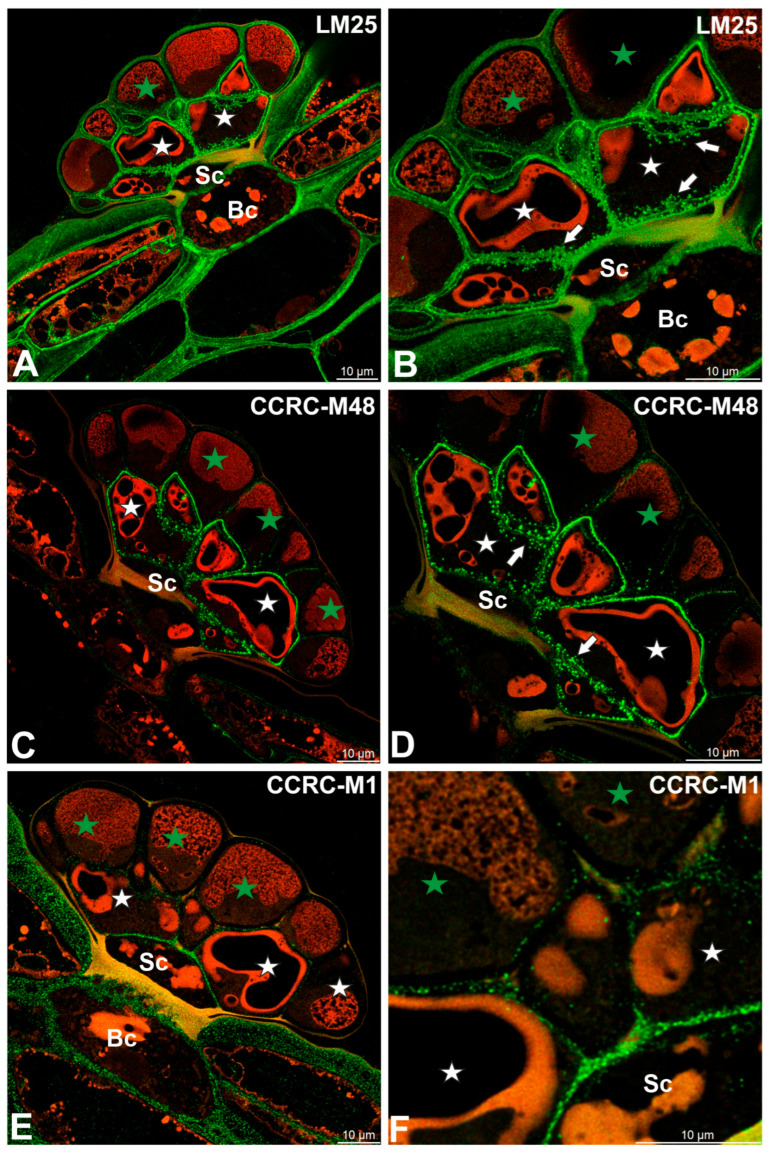
Hemicelluloses detected in the digestive gland of the *Dionaea muscipula*, (intense green color—signal of antibody, red-brown color—autofluorescence) basal cell (Bc), stalk cell (Sc), secretory cells (white star) of the inner layer, glandular cells of the outer layer (green star) cell wall ingrowths (white arrows). (**A**,**B**) Labeling with LM25 antibody (which recognizes land plants galactoxyloglucans). (**C**,**D**) Labeling with CCRC-M48 antibody (which reacts with xyloglucan and recognizes the XXLG, XLLG glycan group of non-fucosylated Xyloglucan-5). (**E**,**F**) Labeling with CCRC-M1 antibody (xyloglucan).

Fluorescence labeling with CCRC-M138, which recognizes the glycan group of xylan-6, was detected in cell wall ingrowths as well as in the inner regions of the cell walls of secretory cells (Figure 5A). A broadly similar distribution pattern was observed for xyloglucan epitopes recognized by the LM15 antibody (XXXG motif), which were present in the cell walls of all cell types within the digestive glands, with particularly strong labeling associated with cell wall ingrowths (Figure 5B). Likewise, galactoxyloglucan epitopes detected by LM25 were distributed throughout the cell walls of all gland cell types, again showing pronounced enrichment in cell wall ingrowths (Figure 6A,B).

Immunolabeling with the CCRC-M48 antibody, which recognizes non-fucosylated xyloglucan epitopes (XXLG, XLLG), revealed strong fluorescence signals in cell wall ingrowths and in the cell walls of secretory cells from the inner head layer (Figure 6C,D). In contrast, only weak labeling was detected in the cell walls of secretory cells from the outer head layer (Figure 6D).

A distinct distribution pattern was observed for fucosylated xyloglucan epitopes detected using the CCRC-M1 antibody. In this case, fluorescence signals were predominantly localized to the cell walls of basal and stalk cells, with the exception of walls impregnated with cutin (Figure 6E,F). Only faint labeling was observed in the cell walls of secretory cells from the inner head layer (Figure 6E,F).

### 2.4. Histochemistry Staining (Dye Staining)

**Figure 7:**
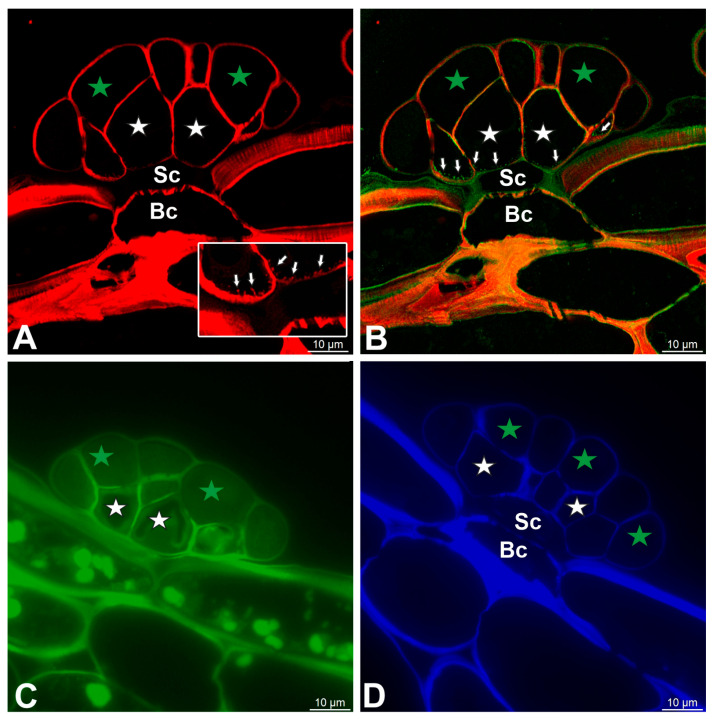
Dye staining of the glands, basal cell (Bc), stalk cell (Sc), secretory cells (white star) of the inner layer, and glandular cells of the outer layer (green star). (**A**) A section of the gland stained with Carbotrace 680 (red color) panel—magnification showing cell wall ingrowths (arrows). (**B**) Double labeling of cells with Carbotrace 680 (red color) and LM15 antibody (green color) to show occurrence of cell wall ingrowths (arrows). (**C**) A section of the gland stained with Carbotrace 520 (green color). (**D**) A section of the gland stained with Calcofluor White (blue color).

The cell walls of gland cells were intensively stained with Carbotrace 680, except for cutin-impregnated cell walls (Figure 7A,B). Compared to cell walls, wall ingrowths showed a very weak signal (Figure 7A,B). The cell walls of gland cells were stained with Carbotrace 520 (Figure 7C). The cell walls of gland cells were intensively stained with Calcofluor White, except for a cutin-impregnated region (Figure 7D).

## 3. Discussion

### 3.1. Plant Cell Wall Microdomains: Concept

Plant primary cell walls are not homogeneous matrices but mosaics of spatially and compositionally distinct areas that underpin local mechanical properties and developmental outcomes such as tip growth, pavement cell morphogenesis, Casparian strip formation, and mucilage release [70,71]. Dauphin et al. [71] proposed the term “cell wall microdomains” to describe these specialized areas. A cell wall microdomain is defined as a subcellular wall territory with a discrete molecular signature—specific degrees of homogalacturonan (HG) methyl-esterification, local enrichment or depletion of hemicelluloses and cellulose, and the targeted deposition or activation of oxidative enzymes—that together tune local softening, loosening, or stiffening of the wall [66,71].

Two recurrent and interdependent modules are central to microdomain formation and remodeling. First, finely patterned HG demethylesterification mediated by pectin methylesterases (PMEs) and their inhibitors (PMEIs) establishes “bar codes” of pectin chemistry that modulate Ca^2+^ crosslinking (egg-box formation) or expose substrates for polygalacturonases and pectate lyases, thereby biasing a microdomain toward stiffening or depolymerization and softening, respectively [71,72]. Second, localized action of oxidative enzymes, notably class III peroxidases (CIII PRXs) and laccases, drives polymerizing outcomes (lignification or extensin crosslinking) or depolymerizing outcomes (ROS-mediated cleavage) depending on enzyme identity, substrate availability, and the redox microenvironment [71,73,74,75]. Importantly, HG demethylesterification patterns can serve as anchoring platforms for specific PRXs; for instance, PMEI6-mediated HG patterning directs PRX36 anchoring to drive mucilage microdomain loosening, while membrane proteins such as CASPs define plasma membrane scaffolds that recruit oxidases and direct lignification in the Casparian strip [71,76,77]. Cell wall microdomains were found in the glands of carnivorous plants, such as *Utricularia*, in terms of arabinogalactans, pectins, and hemicelluloses [78,79].

### 3.2. Pectic Homogalacturonans (HGs) and Other Pectic Polysaccharides

Polysaccharide-rich plant cell walls are hydrated, especially pectins, which are identified as the most hydrated and dynamic polysaccharide component of the primary cell wall. Their hydration strongly influences the mobility and accessibility of the wall matrix. The higher affinity of water for pectins supports the idea that pectins play a central role in controlling wall porosity and diffusion properties, serving as a hydrated matrix in which cellulose microfibrils and hemicelluloses are embedded [80]. Płachno et al. [42] applied the PAS reaction to cell walls of *Dionaea muscipula* digestive glands. A positive result of this reaction indicates that the cell walls of these glands contain polysaccharides. However, this reaction is not selective, indicating the presence of both pectins and hemicelluloses, as well as neutral polysaccharides.

We found that the location of glandular cells affected the composition of cell walls in the cases of low-methylesterified and fully de-esterified HGs. Glandular cells from the outer head layer were deficient in these pectic homogalacturonans, in contrast to cells from the inner head layer. Cell walls enriched in low-methylesterified homogalacturonan (LM-HG) exhibit distinct physicochemical and structural properties that influence wall mechanics and intercellular adhesion. When de-esterification of homogalacturonan occurs in a blockwise manner, contiguous stretches of galacturonic acid residues are capable of coordinating Ca^2+^ ions, resulting in the formation of Ca^2+^-pectate crosslinked gels. These crosslinks substantially increase wall stiffness and rigidity while reducing local extensibility, thereby limiting cell expansion in the affected wall regions [81]. Furthermore, LM-HG accumulation enhances cell–cell adhesion and structural cohesion between adjacent cells. This property plays a crucial role in maintaining tissue integrity [82]. In the case of glands, the properties of low-methylesterified homogalacturonan may be important in secretory cells with an internal layer, as these cells mediate the connection between external secretory cells and stalk cells.

In *Aldrovanda vesiculosa*, the cell walls of stalk and head cells were deficient in HG epitopes recognized by JIM5; however, low-methyl-esterified HGs (recognized by LM19) were present in the gland cells [67]. In *Drosophyllum lusitanicum*, the mucilage glands of secretory cells were deficient in HG epitopes [83].

The β-1,4-galactan (recognized by the LM5) is a well-characterized pectic epitope that occurs predominantly in the cell walls of mature and differentiated plant cells. It is especially abundant in tissues undergoing secondary modifications or fulfilling mechanical and conductive functions, such as sclerenchyma fibers, vascular tissues, and collenchyma [84,85,86,87]. This distribution pattern indicates that β-1,4-galactan contributes to the structural reinforcement and physiological specialization of plant cell walls. We found differences in the occurrence of β-1,4-galactan between the secretory cells of the head. This corresponds to the occurrence of low methylesterified and fully de-esterified HGs. The presence of LM5 is typical for primary, extensible, hydrated walls. β-1,4-galactans contribute to cell wall flexibility and hydration by maintaining spacing between cellulose microfibrils and providing a hydrated matrix within the RG-I domain [88]. Hence, LM5-positive walls are typically more elastic and hydrated, whereas LM5-negative walls are stiffer, enriched in hemicellulose and lignin, and less hydrated. In the outer head cells, the galactan chains have been enzymatically degraded or masked. However, to determine this, it is necessary to trace the development of the glands. It should be added that the absence of LM5 labeling does not always mean the absence of β-1,4-galactan. The epitope can be masked by other wall components such as arabinan, methyl-esterified homogalacturonan, or tightly associated proteins, preventing antibody access [89].

Unlike in secretory cells of glands, we observed pectic polysaccharide alpha-1,5-arabinan (recognized by the LM6) in the cell walls of epidermal and parenchyma cells. Only basal cells of glands had this polysaccharide in their walls. Immunolocalization studies show LM6 labeling in cell corners, middle lamellae, and primary walls of flexible tissues, particularly in epidermal and parenchyma cells of leaves and fruits [90,91]. Noteworthy is the accumulation of β-1,4-galactan in the cell walls of trap epidermal and parenchyma cells. This may suggest the importance of galactan in the mechanics of the trap, but this topic requires further research.

In summary, in terms of the occurrence of pectic homogalacturonans and other pectic polysaccharides, the cell walls of secretory cells from different layers constitute microdomains of the cell wall in the digestive glands of *Dionaea muscipula*.

### 3.3. Hemicelluloses

We are the first to show the diverse distribution of hemicellulose in the digestive glands of *Dionaea muscipula*. We found a similar pattern of xyloglucan and galactoxyloglucan distribution after using LM15 and LM25 antibodies. A slightly different pattern was observed for xylan, which was mainly found in cell wall ingrowths.

However, the use of CCRC-M48 antibody showed that head cells are not equivalent in terms of the occurrence of epitopes recognized by this antibody (the XXLG, XLLG glycan group of non-fucosylated xyloglucan-5). Similarly, CCRC-M1 antibody labeling showed differences in epitope (fucosylated xyloglucan) occurrence between gland cells.

Therefore, we can discuss the formation of microdomains in relation to the presence of hemicellulose.

What could be the role of xyloglucan and galactoxyloglucan in glands? Xyloglucan is the major hemicellulosic polysaccharide of the primary cell wall in most dicotyledonous and non-commelinid monocotyledonous plants [92]. This structural arrangement enables xyloglucan to interact non-covalently with cellulose microfibrils, forming a flexible, hydrogen-bonded network that maintains wall integrity while allowing for controlled extensibility [65,93]. In the cell wall architecture, xyloglucan acts as a molecular tether between adjacent cellulose microfibrils, a concept long known as the “tethered network” model [54,94]. Through this crosslinking, xyloglucan regulates the balance between wall strength and plasticity. Its enzymatic modification, particularly by xyloglucan endotransglucosylase/hydrolases, facilitates wall loosening during cell elongation and expansion [95,96], which could be important during secretion and absorption by gland cells. Galactoxyloglucan, a structurally related but more heavily galactosylated derivative of xyloglucan, contributes to the fine-tuning of wall properties in certain tissues. The galactosylation level affects the solubility of xyloglucan, its interaction with cellulose, and its susceptibility to enzymatic cleavage [97,98]. Highly galactosylated xyloglucans are typically found in maturing or specialized tissues, where they provide increased flexibility and hydration capacity, supporting cell wall plasticity under varying mechanical and osmotic conditions [99]. In contrast, walls containing less substituted xyloglucans tend to be more rigid and mechanically resistant.

It is worth noting that the pattern of occurrence of galactoxyloglucan (recognized by the LM25 antibody) and xyloglucan (recognized by the LM15 antibody) differs from the pattern of occurrence of arabinogalactan proteins in the cell walls of the digestive glands of *D. muscipula* [67].

### 3.4. Cell Wall Ingrowths

There are differences in the presence of specific pectic homogalacturonans (epitopes recognized by antibodies JIM5 and CCRC-M38) in cell wall ingrowths. Thus, in terms of the occurrence of pectic homogalacturonans, cell wall ingrowths constitute microdomains of the cell wall. It is interesting to note two “populations” of cell wall ingrowths in secretory cells. When we used the JIM14 antibody (which labels AGPs), cell wall ingrowths were revealed in both the outer and inner layers of cells. However, when we used the CCRC-M38 antibody in the same cells, positive labeling was only observed in cell wall ingrowths in cells from the inner layer. HGs (detected by JIM5) were not present in the cell wall ingrowths in the *Aldrovanda vesiculosa* gland cells [67]. De-esterified HGs were absent from the wall ingrowths in transfer cells of *Elodea canadensis* leaves [100]. Both methylesterified and de-esterified HGs occurred in the cell wall ingrowths in transfer cells of *Phaeoceros carolinianus*, *P. laevis* [101], *Physcomitrium patens* [102], and *Marchantia polymorpha* [103]. Our work demonstrated that the hemicelluloses recognized by the CCRC-M138 antibody (for xylan), LM25 antibody (for galactoxyloglucan), LM15 antibody (for xyloglucan), and CCRC-M48 antibody (for xyloglucan) were present in the cell wall ingrowths in cells of *Dionaea muscipula* secretory cells. Hemicelluloses were also detected in cell wall ingrowths in gland cells of *Aldrovanda vesiculosa* [67] and *Drosophyllum lusitanicum* [83]. Xyloglucan and galactoxyloglucan were recoded in cell wall ingrowths in bryophyte transfer cells [101,102,103].

In *Dionaea muscipula*, cell wall ingrowths were not stained with Carbotrace 520 and Calcofluor White. After staining with Carbotrace 680, wall ingrowths showed a very weak signal. This indicates that cell wall ingrowths in *Dionaea* contain cellulose in such small amounts (compared to the cell walls) that we had difficulty detecting it. This is interesting because in some plant species, cellulose synthesis is a key early factor in the construction of “reticulate” wall ingrowths [104]. Additionally, in the digestive glands of *Aldrovanda vesiculosa*, Calcofluor White staining revealed the presence of cellulose in the cell wall ingrowths [67].

## 4. Materials and Methods

### 4.1. Plant Material

*Dionaea muscipula* J. Ellis traps were obtained from the first author’s collection; the plants were grown in a humid terrarium at room temperature. A mixture of acidic peat and sand was used as the substrate. The pots stood in water. In total, 2–3 glands were inspected across three independent plants (biological material).

### 4.2. Immunochemical Analysis

The traps were cut into small fragments and fixed as described by Płachno et al. [105]. For analysis of the occurrence of the major cell wall polysaccharides and glycoproteins, the plant material was dehydrated with acetone and embedded in an Epoxy Embedding Medium Kit (Fluka, Seelze, Germany). Sections were cut on a Leica Ultracut UCT ultramicrotome (Leica Biosystems, Wetzlar, Germany, 0.7 µm thick). The rehydrated sections in PBS buffer were blocked with 1% bovine serum albumin (BSA, Sigma-Aldrich, St. Louis, MO, USA) in a PBS buffer and incubated with the following primary antibodies overnight at 4 °C: anti-homogalacturonans (HGs): JIM5 (low methylesterified HGs), JIM7 (highly esterified HGs), LM19 (low methylesterified HGs), and CCRC-M38 (fully de-esterified HGs); anti-hemicelluloses: LM25 (galactoxyloglucan), CCRC-M48 (xyloglucan, antibody recognizes the XXLG, XLLG glycan group of non-fucosylated Xyloglucan-5), CCRC-M1 (xyloglucan, antibody recognizes the alpha-Fuc-(1,2)-beta-Gal glycan group of fucosylated xyloglucan), LM15 (xyloglucan, antibody recognizes the XXXG motif of xyloglucan), CCRC-M138 (xylan), e.g., [89,106,107]; anti-arabinogalactan protein: JIM14. All primary antibodies were used at a 1:20 dilution. They were purchased from Plant Probes, Leeds, UK (rat monoclonal antibodies: JIM5, JIM7, LM19, LM25, LM15, JIM14) and Agrisera, Vännäs, Sweden (mouse monoclonal antibodies: CCRC-M38, CCRC-M1, CCRC-M48, and CCRC-M138). Secondary antibodies goat anti-rat secondary or anti-mouse antibody conjugated with FITC (fluorescein isothiocyanate) [excitation wavelength (λEx): ~495 nm, emission wavelength (λEm): ~519 nm] or Alexa Fluor 488 [excitation wavelength (λEx): 495 nm, emission wavelength (λEm): 519 nm], respectively, were purchased from Abcam (Cambridge, UK). The samples were then cover-slipped using a Mowiol mounting medium: a mixture of Mowiol ^®^4-88 (Sigma-Aldrich) and glycerol for fluorescence microscopy (Merck, Warsaw, Poland) with the addition of 2.5% DABCO (Carl Roth GmbH + Co. KG, Karlsruhe, Germany). They were viewed using a Leica STELLARIS 5 WLL confocal microscope (Leica Biosystems, Wetzlar, Germany) with Lightning module deconvolution. Confocal imaging was performed on a Leica system equipped with a White Light Laser (WLL) using a Leica HC PL APO 63×/1.40 oil-immersion objective (Leica Biosystems, Wetzlar, Germany). Fluorescence was recorded in two channels: the green channel was excited at 494 nm (WLL; laser intensity 8.34%; detector gain 2.5), and the red channel was excited at 540 nm (WLL; laser intensity 5.24%; detector gain 35.9). Presented images are maximum projections from acquired z-stacks. Negative controls were created by omitting the primary antibody step, which caused no fluorescence signal in any of the control frames for any stained slides (Figure S1).

### 4.3. Histochemical Analysis

Cellulose was labeled using Carbotrace 680 and Carbotrace 520 (Ebba Biotech AB, Nobels väg 16 S-171 65 Solna, Sweden) [108]. Crystalline cellulose was also labeled using Calcofluor White Stain (Merck Life Science Sp.z.o.o., an affiliate of Merck KGaA, Darmstadt, Germany). Sections were viewed using a Leica STELLARIS 5 WLL confocal microscope or a Leica DM6000B microscope equipped with a DAPI (Ex/Em = 350/450 nm wavelength; exposure time, 347.136 ms with gain = 1) and a Rhodamine filter (Ex/Em = 546/585 nm wavelength; exposure time, 661.156 ms with gain = 1.9).

### 4.4. Quantification of Fluorescence Intensity in the Cell Wall and Wall Ingrowth Regions

Relative fluorescence intensity was quantified using region-of-interest (ROI) measurements performed in Leica LAS X (Leica Microsystems, Wetzlar, Germany). For each antibody, two independent control glands were analyzed (biological replicates; n_bio = 2; Rep1 and Rep2). Within each gland, two ROIs were manually delineated for (i) the cell wall region adjacent to wall ingrowths (cell wall ROI) and (ii) the wall ingrowth region (ingrowth ROI) using freehand selection (technical repeats; n_ROI = 2 per region and replicate). ROI areas ranged from 1.75 to 3.00 µm^2^.

All confocal acquisition settings were kept identical across samples and matched those used during image acquisition (including laser power, detector gain, pinhole, and scan parameters), and images were analyzed without altering intensity scaling. For each target ROI, an ROI_correction was recorded in the corresponding local background or cytoplasmic area (as defined in the analysis sheet), and the corrected mean fluorescence intensity was calculated as follows:

MFIcorr (a.u.) = Mean(ROI_target) − Mean(ROI_correction).

For each replicate and region (cell wall and ingrowths), MFIcorr values from the two technical ROIs were averaged to obtain a single replicate-level value, and the technical variability was reported as SDtech calculated from the two technical ROIs (n_ROI = 2). Due to the limited number of biological replicates (n_bio = 2), the dataset is presented as descriptive quantification without inferential statistics. For epitopes without detectable labeling in the wall ingrowth region, the ingrowth ROI corresponded to local background, resulting in MFIcorr values of 0.00.

Descriptive quantification of corrected mean fluorescence intensity (MFIcorr) in the cell wall and wall ingrowth regions in secretory cells of the inner layer is shown in Table S1.

## 5. Conclusions

We found that the cells within the gland head are not equivalent. Their location determines the composition of their cell walls in terms of the presence of various epitopes.

Inner-layer gland cells contain abundant low-methylesterified and de-esterified HGs, which promote Ca^2+^-mediated crosslinking, wall stiffening, and enhanced cell–cell adhesion. Outer-layer cells lack these epitopes, indicating more flexible and dynamic walls adapted to secretion to the external environment.

Differences in β-1,4-galactan distribution (LM5 epitope) further emphasize microdomain heterogeneity: LM5-positive walls represent hydrated, extensible primary matrices, while LM5-negative walls are likely stiffer due to galactan degradation or epitope masking. These patterns underscore the distinct mechanical regimes operating within the gland layers. β-1,4-galactan (LM5 epitope) is largely confined to cell walls of epidermal and parenchyma cells.

Arabinans (LM6) are largely confined to epidermal and parenchyma tissues and are nearly absent from secretory head cells, demonstrating that the mechanical and functional properties of trap tissues differ sharply from those of the glandular apparatus. Hemicelluloses (xyloglucan, galactoxyloglucan, xylan) show unequal distribution across gland cells, providing additional evidence for microdomain formation.

Variability in LM15, LM25, CCRC-M48, and CCRC-M1 labeling indicates that xyloglucan substitution patterns differ between gland layers, likely modulating wall flexibility, hydration, and the mechanics of secretion and uptake.

In terms of the occurrence of pectic homogalacturonans, cell wall ingrowths constitute microdomains of the cell wall in secretory cells of digestive glands.

Taken together, these findings demonstrate that the gland walls of *Dionaea muscipula* form a mosaic of functionally distinct microdomains, shaped by specific combinations of pectins, hemicelluloses, and AGPs. This structural heterogeneity underlies the functional differentiation of gland cells and supports the modern concept of plant cell wall microdomains as applied to carnivorous plant glands. Our results contribute to the current knowledge regarding cell wall structure and cell wall microdomains in carnivorous plant glands.

## Data Availability

The original contributions presented in this study are included in the article/Supplementary Materials. Further inquiries can be directed to the corresponding author.

## Supplementary Materials

The following supporting information can be downloaded at: https://www.mdpi.com/article/10.3390/ijms27031193/s1.
