# Supplementary material for: Immunocytochemical Analysis of the Wall Ingrowths and Cell Wall Microdomains in the Digestive Glands of Venus’ Flytrap
Source: Int J Mol Sci. 2026 Jan 24;27(3):1193. doi: 10.3390/ijms27031193 (PMC12897253; doi:10.3390/ijms27031193)

Figure S1

Figure S1. Control reactions of cell wall components after immunolabeling (green color – signal of antibody; yellow and red-brown colors – autofluorescence), (**A**-**B**). Section through the peltate digestive glands.

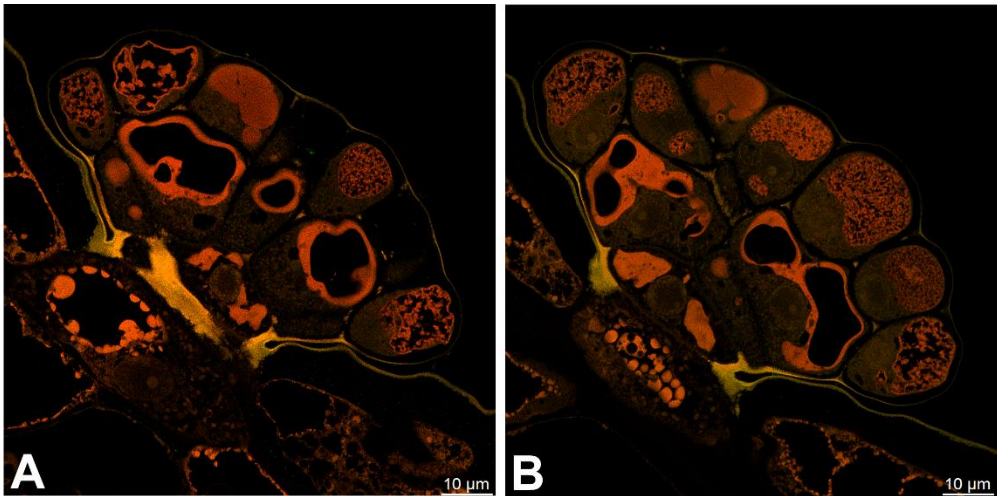

Supplement: Supplementary file 1 [file ijms-27-01193-s001.zip › ijms-4085788-supplementary Figure S1.pdf]
