# Supplementary material for: Immunocytochemical Analysis of the Wall Ingrowths and Cell Wall Microdomains in the Digestive Glands of Venus’ Flytrap
Source: Int J Mol Sci. 2026 Jan 24;27(3):1193. doi: 10.3390/ijms27031193 (PMC12897253; doi:10.3390/ijms27031193)
Supplement: Supplementary file 1 [file ijms-27-01193-s001.zip › ijms-4085788-supplementary Table S1.pdf]

Table S1. Descriptive quantification of corrected mean fluorescence intensity (MFIcorr) in the cell wall and wall ingrowth regions in secretory cells of the inner layer (n_bio = 2). MFIcorr (a.u.) denotes corrected mean fluorescence intensity calculated as ROI_target minus ROI_correction (background/vacuole), as defined in the Methods. Rep1 and Rep2 are biological replicates (two independent control glands; n_bio = 2). SDtech is the technical standard deviation calculated from two ROIs per replicate (n_ROI = 2) for each region (cell wall and ingrowths). For epitopes without detectable wall ingrowth labelling, the "ingrowths" ROI corresponded to local background, yielding MFIcorr = 0.00.

Antibody (epitope class)	Cell wall Rep1 (MFIcorr ± SDtech, a.u.)	Cell wall Rep2 (MFIcorr ± SDtech, a.u.)	Ingrowths Rep1 (MFIcorr ± SDtech, a.u.)	Ingrowths Rep2 (MFIcorr ± SDtech, a.u.)
Pectins - homogalacturonan (HG)				
**JIM5**	11506.80 ± 388.03	6003.32 ± 954.00	0.00 ± 0.00	0.00 ± 0.00
**JIM7**	10101.50 ± 952.02	15051.05 ± 1190.73	0.00 ± 0.00	0.00 ± 0.00
**LM19**	7583.96 ± 1161.02	5315.38 ± 739.97	0.00 ± 0.00	0.00 ± 0.00
**CCRC-M38**	2082.41 ± 324.63	6383.50 ± 746.86	1808.74 ± 9.12	2131.74 ± 612.57
Pectins - rhamnogalacturonan-I (RG-I) side chains				
**LM5**	826.85 ± 44.97	2680.01 ± 868.72	397.19 ± 388.62	676.50 ± 73.54
**LM6**	4825.77 ± 494.23	1522.00 ± 240.55	0.00 ± 0.00	0.00 ± 0.00
Hemicelluloses - xyloglucan (XyG)				
**LM15**	2504.74 ± 161.18	7064.30 ± 1611.02	1201.37 ± 245.54	758.36 ± 288.67
**LM25**	4783.45 ± 0.71	5129.53 ± 711.05	1328.90 ± 71.75	2941.32 ± 387.35
**CCRC-M1**	956.12 ± 144.31	1203.99 ± 34.67	549.46 ± 399.73	283.80 ± 82.86
**CCRC-M48**	6150.17 ± 509.92	12005.40 ± 3128.56	4201.55 ± 1527.45	10106.86 ± 1282.81
Hemicelluloses - xylan				
**CCRC-M138**	11181.87 ± 5541.77	4985.50 ± 2338.66	6768.75 ± 2848.55	4422.69 ± 1254.36
